# Cancer biomarker discovery is improved by accounting for variability in general levels of drug sensitivity in pre-clinical models

**DOI:** 10.1186/s13059-016-1050-9

**Published:** 2016-09-21

**Authors:** Paul Geeleher, Nancy J. Cox, R. Stephanie Huang

**Affiliations:** 1 Section of Hematology/Oncology, The University of Chicago, 900 E 57th Street, KCBD room 7148, Chicago, IL 60637 USA; 2 Section of Genetic Medicine, Department of Medicine, University of Chicago, Chicago, IL 60637 USA; 3 Division of Genetic Medicine, Vanderbilt University, Nashville, TN USA

## Abstract

We show that variability in general levels of drug sensitivity in pre-clinical cancer models confounds biomarker discovery. However, using a very large panel of cell lines, each treated with many drugs, we could estimate a general level of sensitivity to all drugs in each cell line. By conditioning on this variable, biomarkers were identified that were more likely to be effective in clinical trials than those identified using a conventional uncorrected approach. We find that differences in general levels of drug sensitivity are driven by biologically relevant processes. We developed a gene expression based method that can be used to correct for this confounder in future studies.

**Electronic supplementary material:**

The online version of this article (doi:10.1186/s13059-016-1050-9) contains supplementary material, which is available to authorized users.

## Background

Personalized cancer medicine promised the ability to improve cancer treatment using molecular marker(s) (e.g. genome sequence, gene expression) obtained from the patient’s tumor. There have been some notable successes, for example, tyrosine kinase inhibitors in BCR-ABL1 positive chronic myeloid leukemia (CML) [1]. However, many other compounds/targets have proved ineffective in clinical testing, resulting in financial and human cost. Many studies have also proposed biomarkers aimed at repurposing or improving the efficacy of existing drugs, but there have been countless failures when predictions from pre-clinical data have been applied in the clinic. Overall, the number of clinically applied biomarkers has been described as “staggeringly small” compared to the number proposed in the literature [2]. Thus, there is an urgent need to improve biomarker discovery strategies.

Multi-drug resistance (MDR) is commonly observed in clinical oncology. These are mechanisms that cause cancer cells to develop resistance to many drugs [3]. A canonical example is the upregulation of ABCB1 (also known as multi-drug resistance protein 1 (MDR1)), an efflux protein involved in removing foreign substances (including drugs) from cells. There are many other known mechanisms of MDR, including insensitivity to drug induced apoptosis, activation of pro-survival pathways, and altered tumor permeability [3–5].

In drug development and repurposing, most biomarkers are initially identified through cell line drug sensitivity screening, due to established methods and comparatively low cost [6]. The largest publicly available cell line pharmacogenomics studies to date were screened by the Cancer Genome Project (CGP; sometimes also referred to as the Genomics of Drug Sensitivity in Cancer (GDSC)) and the Cancer Cell Line Encyclopedia (CCLE); both screened panels of approximately 700 cell lines for sensitivity to 138 and 24 compounds, respectively, along with collecting extensive genomic and gene expression data [7, 8]. Additionally, a more recent study, the Cancer Therapeutics Response Portal (CTRP) performed drug sensitivity screening of 481 drugs on the CCLE cell lines [9, 10]. In this study, we show using these large cell line datasets that variability in general levels of drug sensitivity (GLDS) in pre-clinical data confounds biomarker discovery. We have primarily focused on CGP for discovery and CCLE/CTRP for validation and comparison. We present data that suggests that GLDS is likely related to MDR in clinical oncology (although we introduce the term “GLDS” to avoid claiming that these are necessarily identical phenomena). Accounting for the confounding effect of GLDS improves power to discover aberrations truly relevant to drug response and identifies false-positive associations. These findings are highly relevant to biomarker discovery for existing drugs and in cancer drug discovery screens, such as those often employed by large pharmaceutical companies.

## Results

### Variability in general levels of drug sensitivity (GLDS) is evident in cancer cell lines

**Fig. 1:**
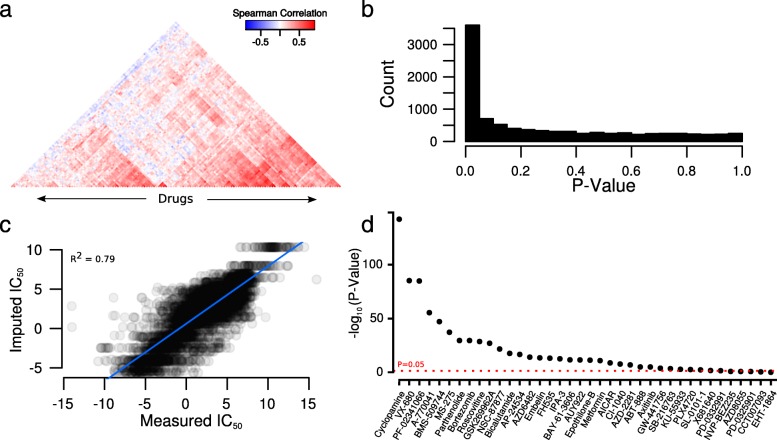
General levels of drug sensitivity can be estimated in a very large set of cell lines. **a** *Heatmap* showing pairwise correlations between IC_50_ values of all drugs in CGP. Drugs are arranged (by Euclidean distance) on the *x-axis*. A full *heatmap* with visible drug names and drug class labels is provided in Additional file 2: Figure S1. **b** *Histogram* of *P* values for pairwise correlation between all 138 drugs in CGP. **c** *Scatterplot* of imputed against measured IC_50_ values from eight-fold cross-validation in CGP. Imputed values were estimated using our iterative matrix completion algorithm. **d** *P* values of the Spearman correlation of the IC_50_ values of 38 randomly chosen drugs against the first principle component of the IC_50_ values of the remaining 100 drugs in CGP. Values above the *dashed red line* have *P* < 0.05

To assess whether GLDS varies in pre-clinical models, we used cell line data from the CGP. First, we performed pairwise correlation between the half maximal inhibitory concentration (IC_50_) values of all 138 drugs across all 714 cell lines. There was a clear pattern whereby some cell lines were sensitive to many drugs, or resistant to many drugs; but only moderate evidence of similar classes of drugs clustering together (Fig. 1a, Additional file 1: Table S1 and Additional file 2: Figure S1). However, there were far more significant correlations between drug IC_50_ values than expected by chance. In fact, of 9453 possible pairwise correlations 3597 reached a false discovery rate (FDR) < 0.05 and 99 % of these were in a positive direction, showing that the effect of many drugs is much more similar than expected by chance (Fig. 1b). This pattern was even stronger in other large pharmacogenomics cell line screening studies; in CCLE 274 of 276 pairwise correlations reached an FDR < 0.05 and 100 % of these correlations were in a positive direction (Additional file 1: Table S2 and Additional file 2: Figure S2). In the CTRP drug screening data, 77,789 of 115,440 pairwise correlations reach an FDR of < 0.05, with 95 % of these in a positive direction (Additional file 1: Table S3 and Additional file 2: Figure S3). Remarkably, strong correlations were not only observed between drugs within the same class, but also clearly evident between drugs with different mechanisms; examples from CGP include bortezomib, a proteosome inhibitor and entinostat, a histone deacetylase inhibitor (*r*_*s*_ = 0.5, *P* = 1.4 × 10^–8^) or motesanib, an angiokinase inhibitor and cisplatin, a cytotoxic platinating agent (*r*_*s*_ = 0.52, *P* = 2.12 × 10^–9^). These examples are among many such associations evident in CGP, CCLE, and CTRP (Additional file 1: Tables S1–S3) and support the notion of mechanisms of GLDS affecting the level of sensitivity to many drugs in pre-clinical models.

### GLDS in cell lines can be summarized using dimensionality reduction

**Fig. 2:**
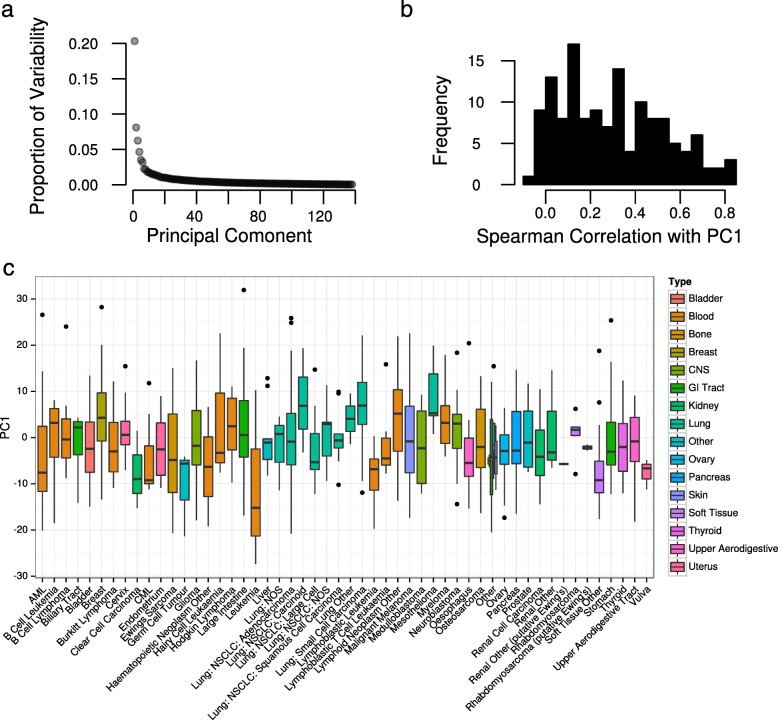
General levels of drug sensitivity in a panel of cancer cell lines. **a** The proportion of variability in the data explained by each of the PCs of the fully imputed drug sensitivity (IC_50_) data matrix in CGP. PCs are arranged by associated eigenvalue. **b** Histogram of Spearman correlations of the IC_50_ values of all 138 drugs in CGP with PC1 of the fully imputed IC_50_ matrix. **c** Boxplot of PC1 (estimated in all 714 cell lines) against tissue-of-origin in CGP. Boxes are colored by cancer type

The pattern of GLDS could be summarized using standard dimensionality reduction techniques; however, to apply such methods, a complete dataset (i.e. matrix of drug IC_50_ values) is required and there were a substantial number of missing values in these datasets (drugs that had not been screened against every cell line). Thus, we applied a custom iterative matrix completion algorithm (see “Methods”) similar to those previously applied to gene expression microarray data [11]. We could impute missing values very accurately, with an improvement observed over previously proposed methods (Fig. 1c, *R*^*2*^ = 0.79 in CGP from eight-fold cross-validation) [12]. With this completed dataset, we summarized the pattern of GLDS using singular value decomposition (SVD). To show that such an approach recapitulates a GLDS signature, we first applied the method to 100 randomly chosen drugs from CGP (i.e. a matrix of IC_50_ values for 100 drugs). The first principle component (PC1) derived from these 100 drugs (across all 714 cell lines) is positively correlated with the IC_50_ values of 33 of the 38 remaining drugs (FDR < 0.05 using Spearman correlation). This demonstrates that this approach is informative towards the response to most additional drugs (Fig. 1d). Hence, we proceeded to apply the approach to all 138 drugs in CGP. PC1of this matrix represents the largest axis of variation in GLDS, explaining 20.3 % of the variability in drug sensitivity (Fig. 2a) and is positively correlated with the IC_50_ values of 114 of the 138 drugs at FDR < 0.05 (Fig. 2b) in CGP. Likewise, 415 of 481 drugs were correlated with PC1 in CTRP and all 24 drugs in CCLE (FDR < 0.05 from Spearman correlation). Thus, this analysis has uncovered a pervasive tendency of cell lines to exhibit sensitivity or resistance to many drugs, regardless of canonical drug mechanisms. As an alternative (and highly interpretable) means to calculate general drug sensitivity, we also calculated the median drug sensitivity value for each cell line across all drugs in CGP, which is unsurprisingly highly correlated with PC1 (Additional file 2: Figure S4, *r*_*s*_ = 0.87, *P* < 2.2 × 10^–16^), demonstrating that this signal can be recovered by independent analytical approaches.

### Finding the biological drivers of GLDS in cell lines

**Fig. 3:**
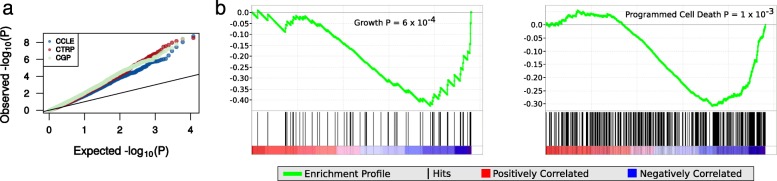
Gene expression and biological processes are associated with GLDS. **a** A *QQ-plot* where the *P* values for an association between gene expression and the first principle component of the completed drug sensitivity matrix are plotted against a theoretical uniform distribution. A deviation from the diagonal line in all three datasets is indicative of an enrichment of low *P* values. **b** GSEA *enrichment plots* for enrichment of Gene Ontology biological processes against GLDS in CGP. Data are shown for two of the most significant processes: “Growth” (*left*) and “Programmed Cell Death” (*right*)

Given that GLDS exists, i.e. that there is a reproducible measurable consistency in the response of cell lines to all drugs in these datasets, an obvious question is whether this phenomenon is an experimental artifact, or whether there appears a (consistent) biological basis for the signal (although we emphasize that in either scenario one should still control for this signal when searching for predictors of drug response – see next subsection). Firstly, tissue-of-origin appears to be a contributor, for example in CGP a linear model fit for PC1 dependent on tissue-of-origin (51 different tissues encoded as a factor) is statistically significant; however achieves an adjusted *R*^*2*^ of only 8 % (Fig. 2c), suggesting that there may be other biological drivers of GLDS. Thus, we investigated whether any of these could be elucidated using gene expression analysis and Gene Set Enrichment Analysis (GSEA). Firstly, in all 3 studies, many genes were significantly associated with PC1 of the drug sensitivity matrix, with 810, 4,680 and 4,457 genes reaching an *FDR* of < 0.05 in CGP, CCLE and CTRP respectively (Fig. 3a). Of these 185 genes were identified in all 3 studies, which is more than would be expected by chance (*P* = 1.3 × 10^-11^), meaning that there is some cross-study consistency among the genes potentially involved in GLDS. If the biology of multi-drug resistance in cancer is reflected in this pre-clinical setting, there are widely accepted processes that we would expect to be associated with GLDS. These are (1) factors related to the accumulation of drugs within cells, for example the expression of drug efflux pump *MDR1*, (2) cell growth rate, because the most broadly accepted reason for the effectiveness of most chemotherapeutics has been the susceptibility of fast growing cells [13]; and (3) the activity level of apoptotic pathways [14].

Canonical multidrug resistance gene *MDR1* is associated with GLDS in all three studies, e.g. it is associated (*P* < 0.05 from Spearman correlation) with six, two, and three of the first ten principal components (PCs) derived from the drug sensitivity matrix in CGP, CCLE, and CTRP, respectively. In order to systematically elucidate the biology of GLDS we performed a GSEA analysis, whereby we investigated the association between Gene Ontology (GO) terms and PCs derived from the drug sensitivity matrices in each of CGP, CTRP, and CCLE; we generated results for each of the first five PCs in each study. Because of the different composition of drugs and cell lines in each study, the same signal is not necessarily represented on the same PC in each study (Additional file 3: Tables S1–S30). Strikingly, among the most strongly associated processes in CGP were cell cycle, growth, and apoptosis (Additional file 3: Table S1, Fig. 3b), which is an interesting result as most conventional chemotherapeutics are believed to be effective by targeting fast dividing cells [13]. Another widely cited theory is that generally chemo-sensitive cancer cells are those that are primed for apoptosis [14]. Our results suggest that high baseline expression of genes involved in both of these processes are indeed important; however, neither is likely sufficient to fully predict general effectiveness of chemotherapeutics alone. These associations were also identified as nominally significant in CTRP, whereby GO process “Growth” is associated with PC2 (*P* = 0.03) and “Regulation of Apoptosis” is associated with PC3 (*P* = 0.01) and PC5 (*P* = 0.01). Furthermore, in CTRP the strongest association with drug resistance (on PC1) is for “Lipid Transporter Activity.” This gene set contains several ABCB transporter genes and is a process thought to play a role in drug efflux (and thus resistance) [15]; this process was also associated with PC2 in CGP (*P* = 0.05). Finally, included among the GO terms most strongly associated with drug sensitivity in both CTRP and CGP are processes related to transcription and RNA processing and splicing; while it is not immediately obvious why such processes may be strongly associated with GLDS, we have noted that a previous GO analysis of NCI60 cell lines against a growth rate phenotype [16] reveled a very strong enrichment of similar processes (e.g. RNA processing *P* = 1.6 × 10^–64^ and RNA splicing *P* = 1.2 × 10^–46^), suggesting that the enrichment observed in these processes may simply be a proxy for growth rate in cancer cell lines. These results are suggestive of (at least in part) a biological basis for GLDS, but it seems that this basis is complex and is likely affected by many processes and pathways; thus we caution against over-interpreting any single association or claiming an overly-simplistic biological mechanism (particularly from CCLE as the results were derived from only 24 drugs – thus we have not discussed these in detail). Overall however, these findings were broadly consistent with canonical mechanisms of multi-drug resistance, in that growth rate, apoptosis, and mechanisms affecting drug accumulation/efflux exhibit the strongest associations; but most importantly, these results suggest that GLDS is influenced by factors intrinsic to the cancer cells themselves, rather than extrinsic factors or an experimental artifact.

### Conditioning on GLDS in pre-clinical data improves biomarker discovery

We strongly emphasize that there are two crucial reasons why controlling for GLDS in the pre-clinical biomarker discovery phase will improve clinical translation. First, cancer drug biomarkers, discovered in pre-clinical data, are often subsequently tested on relapsed patients, who have undergone multiple rounds of chemotherapy and developed resistance to many drugs (i.e. high levels of MDR). We showed above that patterns of GLDS were evident in pre-clinical data and are likely related to clinical MDR. Thus, the variability in GLDS in pre-clinical data acts as a confounding factor in discovery of biomarkers relevant in the refractory clinical setting. Second, and perhaps even more important, new drugs are often tested in addition to existing standard-of-care multi-drug regimes. In this scenario, only the drug-specific effect, independent of the drugs already used in the treatment, is relevant in predicting response following the addition of a new drug. Controlling for GLDS will allow the identification of drug markers relevant independent of the general effects of chemotherapeutic regimes that are already being used, thus identifying compounds likely to actually improve the effectiveness of existing regimes.

Hence, we describe a method to estimate and remove this “unwanted variability” in GLDS from pre-clinical biomarker-discovery data: for each drug, we selected a set of unrelated drugs as “negative controls” (see “Methods”) and derived a GLDS signature using SVD as described above. We used this conservative approach by employing an orthogonal set of drugs as negative controls to avoid removing drug specific signal; estimating GLDS a single time from the full dataset (e.g. by the median IC50 or PC1) would risk removing a drug-specific signal and thus over-correcting the results and furthermore would not be generalizable to other studies. Conceptually, our proposed approach is similar to methods for removing unwanted variation in gene expression data [17, 18]. Similarly, we controlled for unwanted variation (in GLDS) by including the first ten PCs of the negative control drugs as covariates in a general linear model (see “Methods”). Using this stringent approach, we tested the IC_50_ of each of 138 drugs against the mutation status of the 71 cancer genes sequenced by CGP. Controlling for GLDS has a profound impact on the results. *P* values for some mutation-drug associations fall substantially, while many others improve. Following correction, a total of 210 significant associations (Additional file 1: Table S4) drops to 79 (Additional file 1: Table S5). Sixty-one of these were among the original 210 associations, while 18 were novel (Additional file 1: Table S6). Of note, in the CTRP data the number of biomarkers discovered (at FDR < 0.05) actually increases slightly from 62 to 75. Given that CGP was a slightly smaller dataset but had a larger number of significant associations, we discuss the impact of these analyses on CGP in greater detail below, but also include the top results of the corrected analysis of CTRP data in Additional file 1: Table S7. The results for all associations tested are included in Additional files 4 and 5 for CGP and CTRP, respectively.

**Fig. 4:**
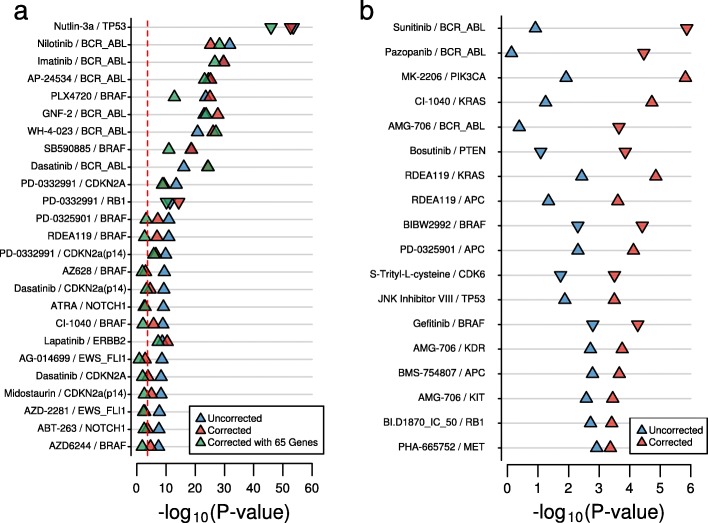
Controlling for general levels of drug sensitivity substantially affects biomarker discovery in the CGP cell lines. **a** *Dot-plot* showing the change in *P* values for the top 25 associations for all sequenced cancer genes across all drugs in CGP. Results are plotted when controlling for GLDS and for an uncorrected approach. Also included are the results when controlling for GLDS estimated from expression data. The *triangle* is pointing in the direction of the effect (i.e. a triangle pointing up indicates a positive effect). **b** A similar *dot-plot* for the 18 novel associations identified in CGP when controlling for GLDS

There were 8881 total mutation-drug association tests conducted on the CGP data. The 25 most significant results were indicative of the effectiveness of this approach (Fig. 4a) and are discussed here in detail; nine of these 25 *P* values improved, 16 become weaker, with four no longer significant at FDR < 0.05. An improved *P* value can be explained by the inclusion of GLDS as a meaningful covariate (thus improving power); whereas a weaker *P* value suggests that the GLDS variable was confounding the original result. Remarkably, in all cases, the associations for which the *P* value improves are supported by existing evidence. For example, six of the top 25 associations were for tyrosine kinase inhibitors (TKI) and BCR-ABL1 positive cell lines. These have arguably been the most successful class of targeted cancer drug in vivo, having transformed the treatment of CML [19]. Tellingly, when we conditioned on GLDS, the *P* values for five of these six associations improved, mirroring the clinical success of these drugs.

Furthermore, a notable improvement in *P* value was observed for lapatinib and ERBB2, a drug which is established as an effective treatment for ERBB2-positive breast cancer patients [20]. Also among the top associations, an improved *P* value was observed for PLX-4720 and BRAF mutation. Supporting this, vemurafenib, which is identical to PLX-4720 except for small modification for pharmacokinetic reasons, was the first BRAF inhibitor approved for the treatment of melanoma patients [21]. A similar improvement was observed for the BRAF inhibitor SB590885; while the effectiveness of this recently developed compound is supported by pre-clinical studies [22], it is yet to undergo clinical trial. Finally, an improvement was observed for PD-0332991 (palbociclib) and RB1 (mutation predictive of resistance). PD-0332991 is designed to inhibit the cyclin D-cyclin-dependent kinases 4 and 6 (CDK4/6)-retinoblastoma (RB) pathway [23]; RB1 expression is an established clinical predictor of sensitivity to this drug, which remains under rigorous clinical investigation in RB expressing breast cancers, where some have reported a doubling of progression-free survival time [24, 25]. These results reveal that inactivating mutations may be equally important in prediction of PD-0332991 resistance.

Equally important, after controlling for GLDS, many mutations that had previously been associated with drug sensitivity were no longer statistically significant. One striking example is that of PARP inhibitors and EWS-FLI1, a relationship that was initially highlighted by the CGP [7]. EWS-FLI1 translocations are characteristic of Ewing’s sarcoma and this putative biomarker was subsequently shown to be an effective predictor in mouse xenograft [26]. Based on these results, PARP inhibitors were tested in patients with refractory Ewing’s sarcoma, who had failed standard chemotherapy. No significant response or durable disease control was observed in the patient cohort [27]. Using a conventional approach, we also found the association of PARP inhibitors in EWS-FLI1 mutation to be among the most significant in these data (*P* = 2.6 × 10^–9^, *P* = 1.9 × 10^–8^; FDR = 1.15 × 10^–6^, FDR = 7.35 × 10^–6^ for rucaparib (AG-014699) and olaparib (AZD-2281), respectively; Fig. 4a). However, when we conditioned on GLDS, these associations were severely affected and for both drugs the association was no longer significant following correction for multiple testing (*P* = 1.3 × 10^–3^, *P* = 2 × 10^–3^; FDR = 0.1 and FDR = 0.13 for rucaparib and olaparib, respectively; Fig. 4a). Thus, these associations were identified because of confounding with GLDS across the biomarker discovery cohort. Conditioning on GLDS predicts the failure of this marker and while other clinical tests of this compound in Ewing’s sarcoma are ongoing, our results do not support any potential benefit in refractory patients. Interestingly, the association between EWS-FLI1 and sensitivity to IGFR inhibitors (OSI-906 and BMS-754807) was also identified using an uncorrected approach; however, these associations actually improved when conditioning on GLDS (Additional file 1: Table S5). The possibility of targeting IGFR in Ewing’s sarcoma has been recognized and even though clinical studies are in the early stage, a complete clinical response of Ewing’s sarcoma following treatment with these compounds has been observed [28, 29]. Thus, we have demonstrated that conditioning on GLDS can not only identify spurious biomarkers, but can also enrich for associations of clinical utility that would otherwise be missed.

Aside from EWS-FLI1 and PARP inhibitors, two other associations among the top 25 were no longer significant (at FDR < 0.05; Fig. 4a) following correction for GLDS, namely BRAF mutation and AZ628 and NOTCH1 mutation and ATRA. To our knowledge, there is no evidence supporting the clinical utility of either of these biomarkers. These observations show that our approach is enriching for biomarkers of clinical utility, which highlights the remarkable potential for this approach to impact personalized medicine.

### Controlling for GLDS identifies new mutation-drug associations

Further interesting associations are those that were identified as significant (FDR < 0.05) when controlling for GLDS, but were not when using an uncorrected approach. In total, 18 novel candidate gene drug associations were identified (Fig. 4b). Among these associations, the BCR-ABL1 fusion gene was predicted to confer resistance to three drugs. These drugs were sunitinib (a receptor tyrosine kinase inhibitor), pazopanib (used to treat a subtype of BCR-ABL-positive cancer albeit with a specific type of mutation), and AMG-706 (an angiokinase inhibitor)). Interestingly, there exists a clinical case report that has documented cases of patients receiving the first of these drugs, sunitinib, actually developing CML [30]. While the possibility of CML developing as a side effect of drug treatment remains speculative, our data are not inconsistent with this observation, although further work will be required to rigorously address this hypothesis.

When conditioning on GLDS a new significant association appears between MK-2206 and PIK3CA mutation (FDR = 5.1 × 10^–4^), a result that is supported by recent phase II clinical studies [31]. CI-1040 sensitivity is now also associated with KRAS mutation, supported by in vitro and in vivo data [32, 33]. Bosutinib is now predicted to be effective against PTEN wild-type cell lines and while this has not been explored in vivo, it has been recently described in pre-clinical data and the mechanism has been rigorously documented [34]. RDEA119 (an experimental MEK inhibitor) is now predicted as effective against KRAS and APC mutated cell lines. A phase II clinical study did not show a difference in response between KRAS mutation and WT pancreatic cancers [35], although this being an experimental compound, this lack of difference could be as a result of lack of efficacy generally; to our knowledge, the effect of APC mutation on RDEA119 sensitivity has not been studied. The remainder of the observed novel associations were primarily for experimental compounds, for which few clinical data have been generated; these associations represent a valuable starting point for follow-up studies.

We have also included results of ElasticNet regression models when GLDS is controlled for, a method often used for constructing predictive models from these types of data (Additional file 1: Table S8); predictors assigned the largest weights were consistent with results obtained when linear regression was used for statistical inference. Finally, we compared the results when tissue or origin was used as a covariate in analyses, instead of GLDS. This is sometimes employed in analysis of large panels of cell lines; however, in any analysis of these or any data obtained from a diverse set of cancers, tissue of origin (or cancer type) will often be highly confounded (and sometimes perfectly confounded) with certain mutations; extreme examples include BCR-ABL1 in CML and EWS-FLI1 in Ewing’s sarcoma, where almost all samples will harbor these aberrations (and other cancer types will not). When tissue of origin is included as a covariate, the number of significant mutation-drug (FDR < 0.05) associations drops to 30 (Additional file 1: Table S9) compared to 79 when GLDS is included instead. Interestingly, for every gene-drug association discussed above that has strong clinical evidence, the *P* value achieved by the GLDS approach is lower than that achieved when instead controlling for tissue of origin, suggesting that GLDS may provide improved power in a diverse set of cancer samples, obviating the need to include tissue of origin as a covariate.

### Controlling for GLDS improves reproducibility between large pharmacogenomics datasets

Reproducibility in large pharmacogenomics screens, specifically between the CCLE and CGP datasets, has been a recent source of debate [36, 37]. Thus, we were interested if controlling for GLDS could also improve concordance between these studies. To test this, we compared gene-drug associations for 15 drugs and 63 sequenced cancer genes common to both CCLE and CGP. Unsurprisingly, conditioning on GLDS improves the reproducibility of findings between these two large studies. Using a naïve approach, 47 % (11 of 23) of mutation-drug associations identified in CGP at FDR < 0.05 were validated at a nominally significant threshold (*P* < 0.05) in CCLE; however, this rises markedly to 62.5 % (10 of 16 remaining significant associations) when we include GLDS as a covariate in the models used to identify these associations in CGP. This finding suggests that variation in GLDS may be at least in part responsible for supposed reproducibility issues in pre-clinical data, as well as clinical data.

### GLDS can be estimated from expression data and used as a covariate in subsequent analysis

The association between expression and GLDS (discussed above) raises a key opportunity to estimate this confounder in a much broader context. It was possible to estimate GLDS in the CGP data because the same cell lines were screened against many drugs. Although data suitable for such analysis likely exists in the drug development realm of large pharmaceutical companies, currently the CGP and CTRP are the only public studies to have performed such a large-scale screening. Hence, our SVD-based approach is not applicable to the majority of conventional biomarker discovery studies. However, measuring genome-wide gene expression using microarrays or RNA sequencing (RNA-seq) is common and straightforward. Thus, using CGP, we have derived a GLDS signature from gene expression microarray data, by identifying the genes most strongly correlated with GLDS (see “Methods”). Unsurprisingly, this set of genes includes canonical MDR genes, such as ABCB6. In CGP, controlling for the first ten PCs of these 65 genes (Additional file 1: Table S10) had a similar effect as controlling for the GLDS principle components derived from drug sensitivity data (Fig. 4a, Additional file 1: Table S11). Controlling for the expression of these genes is somewhat more conservative, as the number of significant associations between drugs and cancer gene mutations drops to 30. However, 25 of these 30 were also among the 79 identified when controlling for GLDS using the method based on the full set of drug sensitivity data. Thus, controlling for these genes dramatically improved the positive predictive value (PPV) from 33.8 % to 83.3 % over an uncorrected approach. This presents the possibility that anybody could estimate the pattern of GLDS in their pre-clinical data without having to screen a vast number of drugs, using gene expression data in the samples under study. If a putative association is robust to controlling for these genes, these results suggest that it is far less likely to be confounded by GLDS, which is a key to successful clinical application. To test the performance of this signature on an external dataset, we applied it to the CTRP data; as above, the first 10 PCs of these 65 genes were calculated from the available gene expression data and included in gene-drug association models as proxy for GLDS. The actual GLDS corrected CTRP data (corrected using the drug data itself, see “Methods”) were treated as a gold standard and the performance of the 65 gene signature was compared to an uncorrected approach. At an FDR of 0.05 the performance increase was moderate; there were 75, 62, and 53 associations in the GLDS corrected (see “Methods”), uncorrected, and expression signature-corrected data, respectively. The PPV in the expression signature-corrected data was 68 %, compared to 62 % for an uncorrected approach. However, upon relaxing the FDR threshold and allowing more associations (thus more power to find agreement/disagreement between the three sets of results), it seemed that the expression signature was performing well, for example at a more liberal FDR of 0.25, we found 390, 760, and 368 associations in the GLDS corrected, uncorrected, and expression signature-corrected data, respectively. A total of 201 and 232 true discoveries were identified by the expression corrected and uncorrected approaches, respectively (again using GLDS-corrected as a gold standard); however, the uncorrected approach identified over three times as many false-positive associations (528 versus 167; PPV 30.5 % versus 54.6 %, *P* = 1.35 × 10^–14^ from Fisher’s exact test). Thus, it is likely that this approach improves discovery on external datasets; however, we stress that this is a conceptually novel class of problem and the community may find ways to improve upon our proposed of solution.

Finally, we investigated whether the set of 65 genes consistently associated with GLDS showed evidence of an association with vital status in 1093 breast cancer samples obtained from the Cancer Genome Atlas (TCGA). Of these genes, 60 were quantified in TCGA RNA-seq data and, indeed, 32 of these were associated with alive/dead status at *P* < 0.05 (from Wilcoxon rank-sum test), which represents a strong enrichment for this set of genes. This suggests that this set of genes, identified in a panel of cell lines, may also be associated with drug response (and hence survival) in an in vivo setting.

## Discussion

Countless proposed cancer biomarkers and novel targeted therapeutics have failed and in this study we have elucidated an important reason why this is the case and developed a widely applicable method to solve this problem. Using the largest available sets of cell line screening data, we found that general levels of drug sensitivity vary in pre-clinical data. Integrative analysis with gene expression data suggests that this phenomenon is likely related to clinical MDR. Many of the biomarkers identified using these cell line data were confounded by this signal, which we have referred to as GLDS. This confounding is highly problematic, because biomarkers (for new or existing drugs) are often tested: (1) on refractory patients (who will typically have developed high levels of MDR); or (2) in conjunction with the current standard of care, whereby only a drug-specific signal is likely to be predictive of response given the addition of the new drug. We showed that it is possible to estimate GLDS in these data and to condition on this signal to identify biomarkers of clinical relevance. Overall, while the reasons for controlling for GLDS are clear, the improved results that we presented were highly predictive of clinical biomarker success, demonstrating the utility of the approach. Indeed, a recent study has also suggested that, similar to GLDS, explicitly controlling for growth rate in these types of assays improved discovery [38]; although our GLDS approach may take this a step further by additionally controlling for other key processes in an unbiased and unsupervised manner. We showed that the expression of various genes, processes, and mutations were associated with GLDS, many of which are also related to clinical MDR; in future it may be possible to derive a model from such a set of mutations and/or expression estimates, which may be useful as an in vivo pan-cancer prognostic indicator. We also showed that it is possible to apply our findings in a broader context, by using our proposed gene expression based signature. This would mean avoiding much of the costs associated with ultimately unsuccessful clinical trials. This study elucidated the nature of GLDS using cell lines; but crucially, relevant biological processes underlie the signal. Thus, a similar bias will almost certainly be evident in other pre-clinical models (e.g. mouse xenografts) and in data derived directly from clinical studies. Larger datasets will allow these hypotheses to be tested for these platforms, but in the meantime these types of studies should carefully consider our findings.

## Conclusion

In conclusion, we have identified variability in GLDS as a novel phenomenon confounding biomarker discovery. We have developed methods to estimate and remove this confounder and overall, these findings can potentially dramatically improve the clinical success rate of drug discovery and repurposing.

## Methods

### Iterative matrix completion algorithm

Drug sensitivity data were obtained from the CGP website (www.cancerrxgene.org), from the CCLE website (http://www.broadinstitute.org/ccle/), and from Additional file 1: Table S3 of the original CTRP publication [9]. Missing drug IC_50_ values were imputed using the following iterative matrix completion algorithm. The IC_50_ values for each of *m* cell lines and *n* drugs is first arranged in an *m* × *n* matrix *X*. The rows of *X* are then ordered (ascending) by the number of missing values. The algorithm is initialized by setting all missing values to the mean IC_50_ value of that particular drug, e.g. all IC_50_ values of cell lines that were not screened with cisplatin are set to the mean value of all cell lines that were screened for cisplatin. Next, we estimated the PCs of *X*. Then, for each missing value of X (*X*_*ij*_) we fit a lasso regression model using the PCs of all other rows of *X* as predictors of all other values (both measured and imputed) for that cell line. The tuning parameter lambda for the lasso regression is selected using cross-validation (implemented in the *glmnet* package in R). The above model is then applied, yielding an updated estimate for *X*_*ij*_. Repeating this procedure for all missing values of *X* yields an updated matrix *X’*. We then estimate *A*, the sum of the total difference between each of the elements of *X* and *X’*. This procedure is repeated iteratively until the total change in *A* converges, which takes approximately 50 iterations. This algorithm offers an improvement over those previously described for expression microarray data; by substituting standard linear regression for lasso regression, we avoid selecting arbitrary numbers of PCs as predictors, because the lasso automatically selects the optimal predictors in the model.

### Controlling for GLDS

For each drug, we selected a set of unrelated drugs as negative controls from which GLDS was estimated. The reason for using negative controls to estimate GLDS, as opposed to the entire dataset, is that using all of the data would also risk removing a drug-specific signal (in addition to GLDS). The negative control drugs were selected based on both their (manually curated) canonical mechanism of action and their empirical correlation in the CGP data itself. For each drug, all drugs with an unrelated mechanism of action (Additional file 1: Table S12) were first selected as negative controls; then, any drugs remaining in this set that were among the 20 in CGP or 100 in CTRP most highly correlated drugs with the drug under scrutiny were also removed from the set of negative controls. This second step is implemented to avoid possible issues with the subjective nature of manual drug classification. It is important to stress that the number of closely correlated drugs to remove is somewhat subjective, and careful consideration needs to be paid to each datasets where this is applied, based on the types of drugs in the dataset, their relatedness and the number of drugs available—the key being to be sure to remove closely related drugs, but to keep enough drugs to still reliably calculate the GLDS signal. Typically, approximately 100 drugs remained as negative controls in CGP and 350 in CTRP. GLDS was then estimated as the first 10 PCs of this set of negative controls. These PCs were then included as covariates in the linear model used to perform the subsequent corrected cancer gene mutation to drug IC_50_ association analysis. Linear models were fit using the standard *lm()* function in R and *P* values were calculated using the *summary()* function, which calculates significance of model coefficients using t-tests. ElasticNet models were fit using the *glmnet* [39] package in R, with an alpha value of 0.5.

### Association of gene expression, GO terms, and gene mutations with GLDS

Gene expression and mutation data were obtained from the CGP website or from the CCLE website. For CGP, gene expression data were preprocessed using the robust multi-array average (RMA) method [40]; for the summarization step, probes were remapped to the latest version of Entrez Gene using BrainArray annotations [41]. The obtained CCLE data were already pre-processed using the BrainArray approach. The association between gene expression levels and the first principle component of the fully imputed drug sensitivity matrix was assessed using a linear model in R. To assess the enrichment of GO terms, first, the potential confounding effect of tissue of origin was first removed by using the residuals of a linear model of the expression of each gene against tissue of origin encoded as a factor. Enrichment of GO biological processes against the first five PCs of the fully imputed drug sensitivity matrix was then assessed using the GSEA software [42]. Genes were ranked by Pearson’s correlation and significance was established using sample label permutations, using at least 1000 permutations.

### Selecting gene expression values as a proxy for GLDS

To identify genes whose expression could potentially be used as proxy for GLDS we performed a Spearman correlation against each of the ten PCs (identified by the method described above) against the expression of every gene. This was repeated for each of the ten GLDS PCs identified for all 138 drugs. For each drug, we compiled a list of the top 50 genes correlated with each PC (i.e. 500 genes for each drug). From this set of genes, we identified 65 genes that were consistently included in this list (i.e. for all 138 drugs). Thus, these genes were always highly correlated with GLDS, regardless of the set of drugs from which GLDS was calculated. The first ten PCs of the expression of these 65 genes were then included as covariates in the linear model used for subsequent corrected gene-drug association analysis.

## Additional files

Additional file 1: Supplementary Tables. (XLSX 3814 kb) Additional file 2: Supplementary Figures. (PDF 1829 kb) Additional file 3: Supplementary Tables from the GSEA analysis. (XLS 2099 kb) Additional file 4: All results from gene-drug association analysis in the CGP data, for an uncorrected analysis, when correcting for GLDS and when correcting for the 65 gene signature. (CSV 1.68 mb) Additional file 5: All results from gene-drug association analysis in the CTRP data, for an uncorrected analysis, when correcting for GLDS and when correcting for the 65 gene signature. (CSV 149 mb)
