# Supplementary material for: Cancer biomarker discovery is improved by accounting for variability in general levels of drug sensitivity in pre-clinical models
Source: Genome Biol. 2016 Sep 21;17:190. doi: 10.1186/s13059-016-1050-9 (PMC5031330; doi:10.1186/s13059-016-1050-9)
Supplement: Additional file 2: — Supplementary Figures. (PDF 1829 kb) [file 13059_2016_1050_MOESM2_ESM.pdf]

Supplementary Figures

June 29, 2016

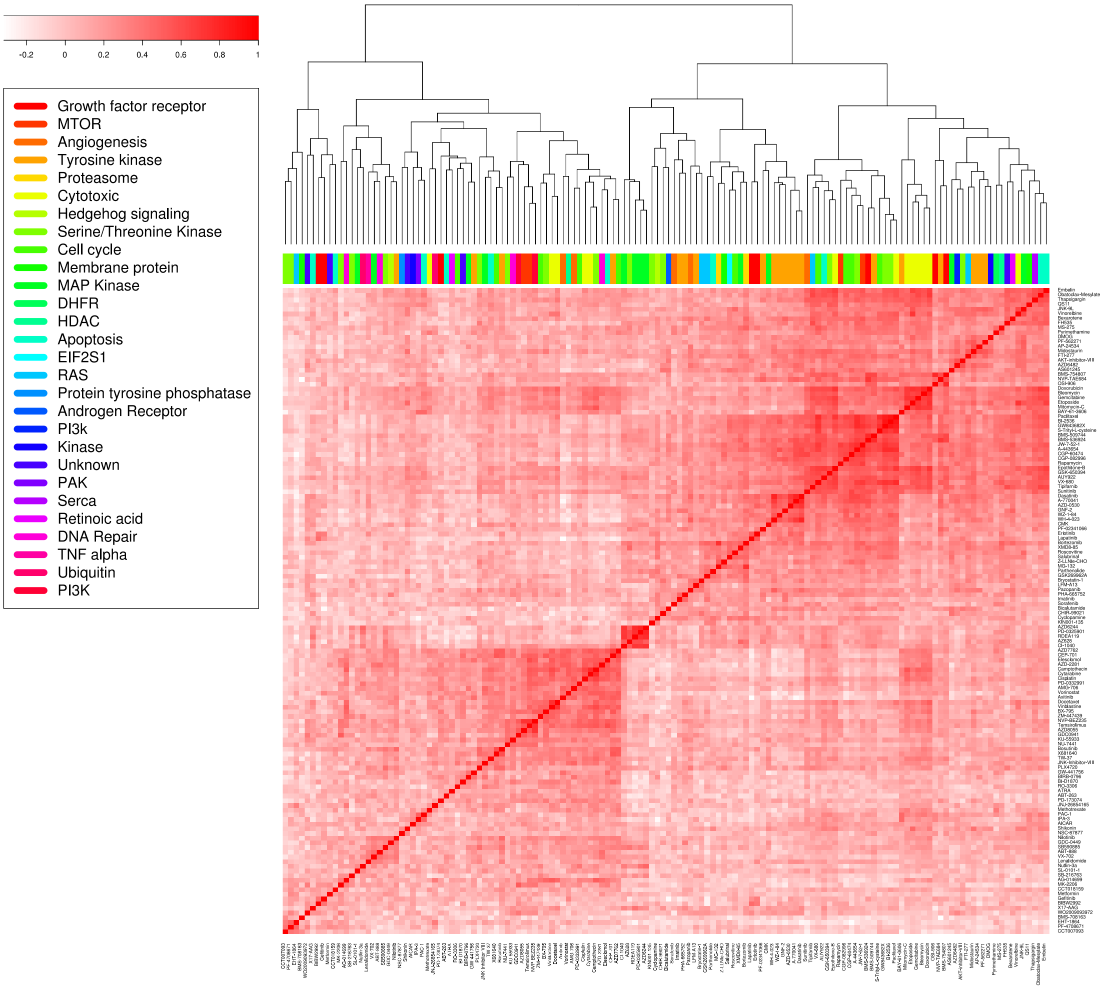

Figure 1: A fully labeled heatmap showing the pairwise Spearman correlations between all 138 drugs in CGP. Drugs are arranged by Euclidian distance.

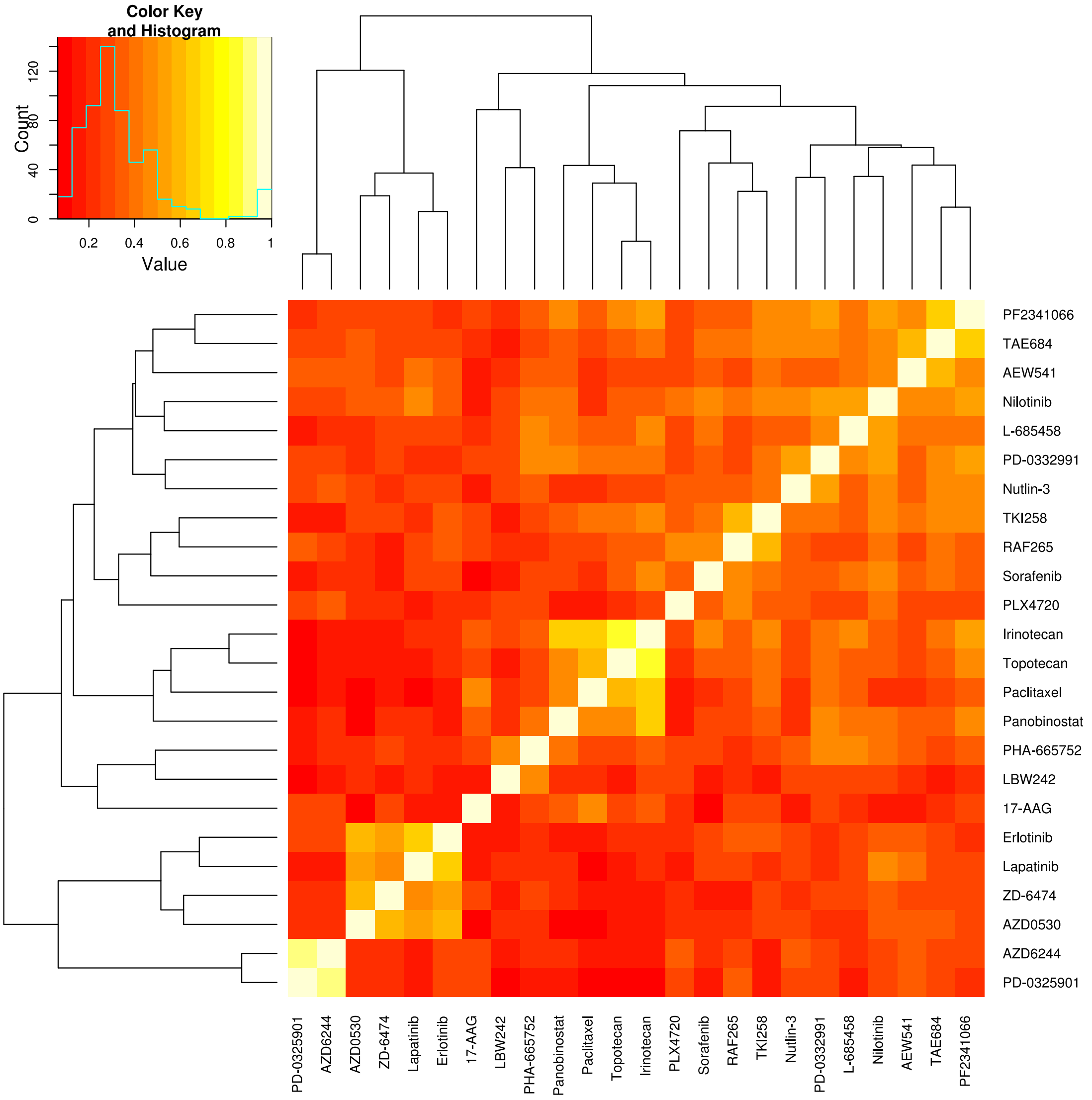

Figure 2: A heatmap showing the pairwise Spearman correlations between all 138 drugs in CGP. Drugs are arranged by Euclidian distance.

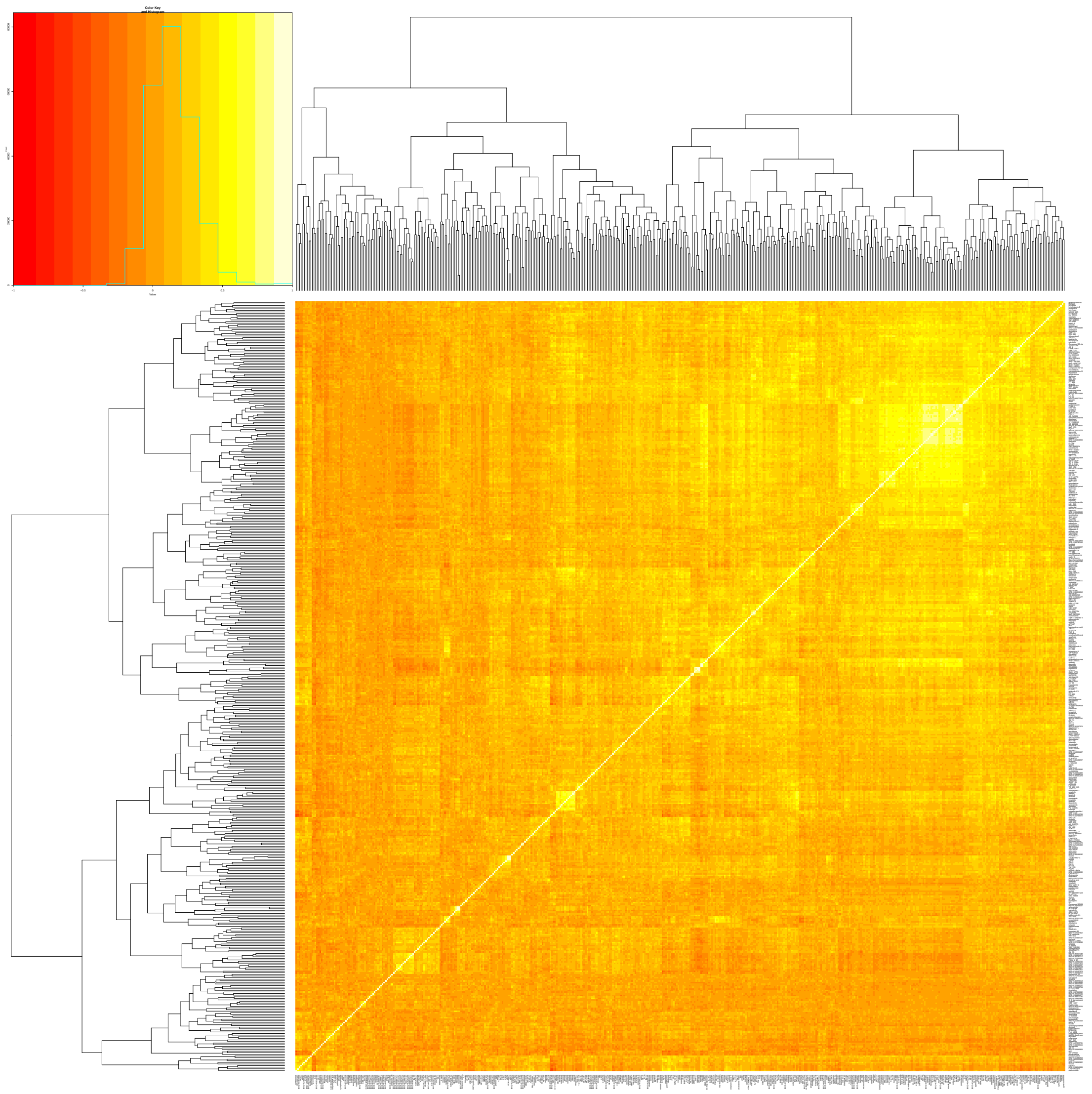

Figure 3: A heatmap showing the pairwise Spearman correlations between all 481 drugs in CTRP. Drugs are arranged by Euclidian distance. This figure contains vector graphics with a lot of detail and is best viewed on a computer screen where the details can be seen by zooming.

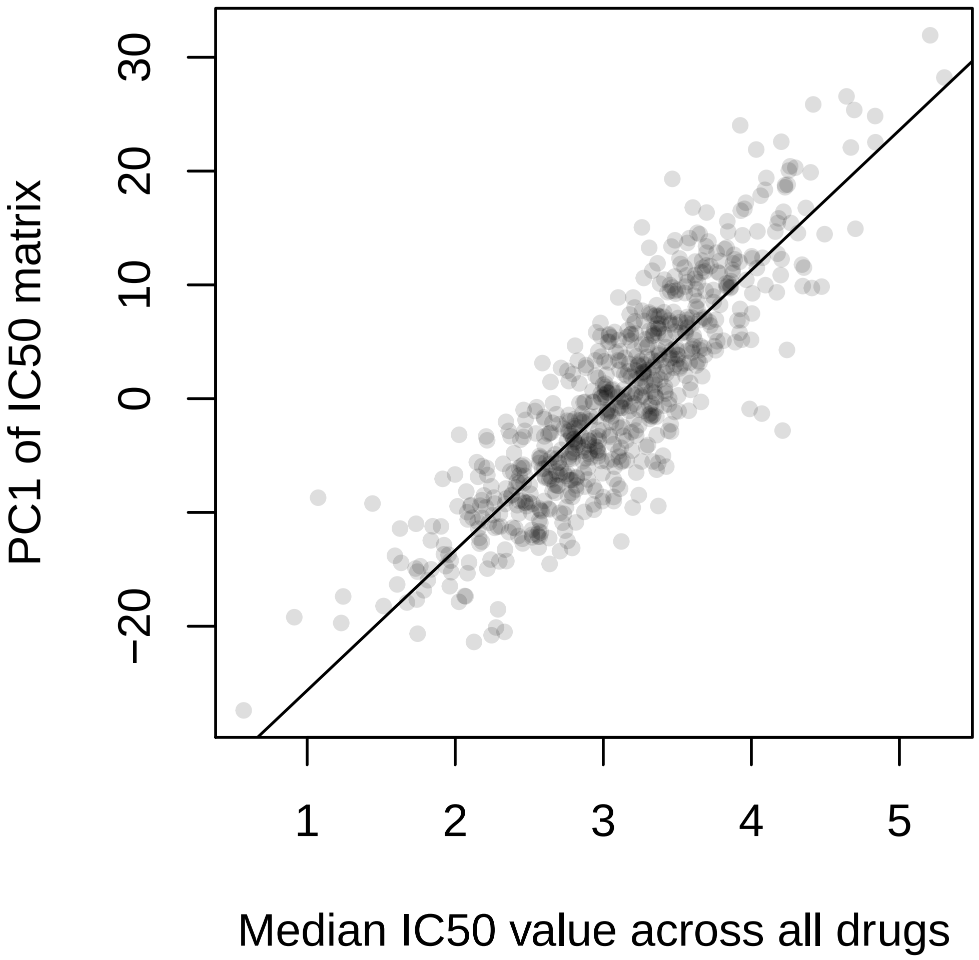

Figure 4: A scatterplot showing (for each cell line) the correlation between the first principle component calculated from the fully imputed IC_{50} matrix and median IC_{50} value calculated across all drugs. These different approaches to calculating a general level of drug sensitivity for each cell line produce highly similar results ($r_s = 0.87$, $P < 2.2 \times 10^{-16}$). Other measures of GLDS, such as calculating the median rank of drug sensitivity (rather than the median IC_{50}) or calculating the mean IC_{50} value also produce almost identical results to those obtained from PC1.
